# Monolithic AgX/Biomass Carbon Aerogels (X = Br, Cl) for Recyclable Photocatalytic Degradation of Multiple Pollutant Classes

**DOI:** 10.3390/gels12080711

**Published:** 2026-08-11

**Authors:** Ziyang Tang, Zhicheng Zhu, Xihao Sun, Yuxin Sun, Bencong Zhang, Mingmei Zhang, Jialu Lu, Wei Wei

**Affiliations:** 1 School of Chemistry and Chemical Engineering, Public Experiment and Service Center, Jiangsu University, Zhenjiang 212013, China; 2 Shanghai Key Laboratory of Special Artificial Microstructure and Technology, School of Physics Science and Engineering, Tongji University, Shanghai 200092, China; jialu21@tongji.edu.cn

**Keywords:** monolithic carbon aerogel, silver halide, electron reservoir, charge separation, multi-class pollutant degradation

## Abstract

While silver halides (AgX) are promising visible-light photocatalysts for water remediation, their practical deployment is severely hindered by intrinsic photocorrosion, rapid charge recombination, and macroscopic recovery challenges. Here, we demonstrate a monolithic AgX/biomass carbon aerogel composite platform, constructed by anchoring AgX nanocrystals in situ onto a 3D hierarchical carbon skeleton. The carbon network not only suppresses nanoparticle aggregation but also plays contrasting optical roles: amplifying the intrinsic visible-light absorption of AgBr while endowing the otherwise UV-confined AgCl with substantial visible-light response. Consequently, the optimal 30 wt% AgBr/CA composite achieves a 95.68% methylene blue degradation efficiency within 60 min—outperforming pristine AgBr by 2.6-fold—while establishing robust activity against two additional, structurally distinct pollutants: rhodamine B and the colorless antibiotic ciprofloxacin. Notably, the free-standing monolith retains exceptional activity over six consecutive cycles. Mechanistic investigations reveal that the carbon aerogel functions as an electron-accepting reservoir, which accelerates interfacial charge separation and steers electron flow toward superoxide radical generation. Notably, XRD and XPS analyses confirm that no detectable metallic Ag^0^ is present in the as-prepared composites. This work establishes a sustainable and scalable architectural paradigm for designing highly efficient, stable, and easily recyclable photocatalytic systems.

## 1. Introduction

The continuous discharge of organic contaminants into natural waters has become one of the most pressing environmental challenges of our time [1,2]. Synthetic dyes released from textile, printing, and dyeing industries, together with antibiotic residues originating from pharmaceutical manufacturing and clinical use, are now ubiquitous in aquatic environments, where their toxicity, chemical persistence, and tendency to promote antibiotic resistance pose serious threats to ecosystems and human health [3,4,5,6]. Conventional treatment technologies, such as adsorption, biological treatment, and membrane filtration, merely transfer these pollutants from one phase to another rather than destroying them. In contrast, semiconductor photocatalysis offers a green and thorough route that exploits solar energy to mineralize refractory organics into innocuous species under ambient conditions, thereby attracting intense interest as a sustainable water-remediation strategy [7,8,9]. Nevertheless, the benchmark photocatalyst TiO_2_ is active only under ultraviolet light, which constitutes less than 5% of the solar spectrum, owing to its wide band gap; developing photocatalysts that respond efficiently to visible light thus remains a central objective of the field [10,11].

Silver halides (AgX, X = Cl, Br) have emerged as a promising family of photosensitive semiconductors in this regard. AgBr possesses a relatively narrow band gap and pronounced photosensitivity, rendering it intrinsically responsive to visible light and making it an effective light-harvesting component in composite photocatalysts [12,13]. AgCl, by contrast, has a wider band gap and is essentially a UV-active material, so that its visible-light activity is generally realized only after coupling with light-absorbing or plasmonic partners [14,15]. Despite their attractive optoelectronic properties, the practical deployment of silver halides is plagued by two intrinsic limitations: a severe photo-instability, whereby photogenerated electrons readily reduce surface Ag^+^ to metallic Ag^0^ and corrode the lattice, and the rapid recombination of photogenerated electron–hole pairs, both of which compromise catalytic activity and durability [16,17,18]. Anchoring AgX nanoparticles onto a rationally chosen support has proven to be an effective remedy: immobilizing AgBr on AlMCM-41 molecular sieves or on graphene-based carriers, for instance, markedly improved both the visible-light activity and the stability of the resulting composites [19,20]. The key, therefore, lies in selecting a support that can simultaneously extend light absorption, accelerate charge separation, and stabilize the AgX phase.

Biomass-derived carbon aerogels (CAs) are particularly attractive supports in this context. As lightweight, three-dimensionally interconnected porous materials, carbon aerogels combine an ultralow density, a high specific surface area, abundant surface functional groups, and broadband light absorption, features that simultaneously favour pollutant enrichment and interfacial charge transfer [7,21,22,23]. Producing such aerogels from renewable biomass further confers the merits of low cost, sustainability, and a tunable carbon framework [5,24,25]. Functionally, the conductive carbon network is expected to function as an electron-accepting reservoir that captures photogenerated electrons and suppresses their recombination, while the porous skeleton spatially disperses and immobilizes the semiconductor nanoparticles, restraining both their aggregation and their photo-dissolution. Moreover, the monolithic, water-insoluble nature of the aerogel allows the catalyst to be recovered almost without loss, circumventing the tedious separation that powdery photocatalysts require. These combined attributes make biomass carbon aerogels an ideal scaffold for constructing robust, visible-light-active silver-halide composites. To date, however, AgBr- and AgCl-based carbon-aerogel photocatalysts have not been investigated within a single, directly comparable framework, leaving open the question of whether one carbon scaffold can simultaneously serve two halides with different optical characters. Moreover, three limitations persist across current AgX/carbon systems: (i) Most reported composites—including AgX/graphene aerogels [21]—are powders or mechanically fragile foams that still require centrifugation or filtration for recovery, so the macroscopic separation problem is mitigated rather than eliminated. (ii) The support in most systems is treated as a passive dispersant, and its dual, halide-dependent optical function has not been systematically disentangled. (iii) Performance is almost always benchmarked against dyes, whose intrinsic light absorption confounds the true photocatalytic contribution through dye-sensitization pathways, leaving activity toward colorless, refractory pollutants such as antibiotics largely unquantified.

Herein, we demonstrate a monolithic AgX/biomass carbon aerogel (AgX/CA) composite platform that fulfills these criteria. Synthesized via a surfactant-directed in situ co-precipitation and freeze-drying route, the hierarchical 3D carbon skeleton not only prevents the aggregation of AgX nanocrystals but also the hierarchical carbon framework acts as an electron-accepting component that facilitates charge separation and promotes the reduction of dissolved O_2_ to reactive oxygen species, while simultaneously boosting the generation of superoxide radicals for the degradation of structurally diverse pollutants including methylene blue, rhodamine B, and the colorless antibiotic ciprofloxacin. Distinct from prior AgX/carbon photocatalysts, our design (i) yields a free-standing, water-insoluble monolith recoverable without centrifugation; (ii) exploits one renewable winter-melon-derived scaffold that plays contrasting optical roles for the two halides; and (iii) is validated against the colorless antibiotic ciprofloxacin, for which dye-sensitization is excluded, thereby isolating the genuine semiconductor-driven activity. Ultimately, the free-standing nature of the ultralight CA monolith enables near-lossless macroscopic retrieval, establishing a highly efficient and readily recyclable structural paradigm for practical wastewater remediation.

## 2. Results and Discussion

### 2.1. Architectural Design and Micro-Interfacial Anchoring of Monolithic Composites

**Figure 1:**
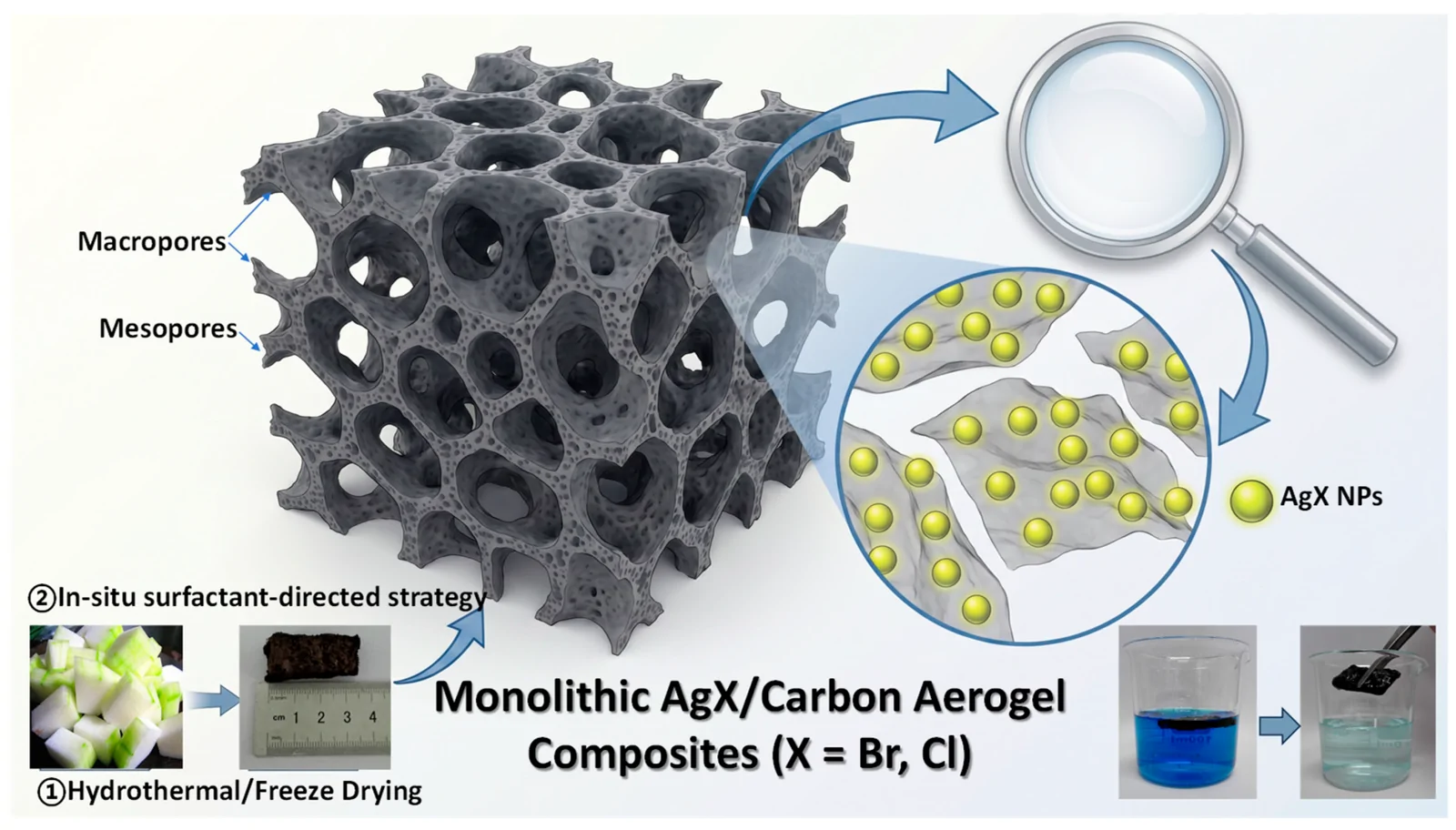
Architectural design and macroscopic features of the monolithic AgX/CA composites.

To circumvent the severe aggregation and formidable recovery challenges typical of powdered photocatalysts, we implemented an architectural paradigm shift by constructing free-standing AgX/carbon aerogel (AgX/CA) monoliths (Figure 1). Winter melon, an abundant and inexpensive biomass with a naturally hierarchical porous tissue, was chosen as the carbon precursor. The soft flesh was cut into cubes, rinsed, and hydrothermally treated at 180 °C for 12 h, converting it into a carbonaceous wet-gel that inherited a three-dimensionally (3D) interconnected network and bore abundant oxygen-containing functional groups on its surface. For the bromide system, the wet-gel was immersed in a CTAB water/ethanol solution, in which the cationic surfactant adsorbed onto the negatively charged carbon surface and simultaneously acted as the bromide reservoir; the CTAB-impregnated gel was then transferred into AgNO_3_ solution, where the captured Ag^+^ reacted in situ with the pre-anchored Br^−^ to nucleate AgBr nanocrystals directly on the gel skeleton. The AgCl/CA composites were prepared through an analogous route in which cetyltrimethylammonium chloride (CTAC) replaced cetyltrimethylammonium bromide (CTAB) as both the surface-directing agent and the chloride source. Finally, freeze-drying removed the entrapped solvent while faithfully preserving the porous architecture, affording monolithic composites with nominal AgX loadings of 10, 20, and 30 wt% (denoted as x wt% AgX/CA).

This in situ, surfactant-directed strategy offers two structural merits. First, the oxygen-containing groups of the carbon aerogel serve as anchoring sites that capture Ag^+^ and direct the homogeneous nucleation of the silver-halide nanocrystals, thereby restraining their aggregation [19,26]. Second, the resulting composites are free-standing, ultralight 3D monoliths: the 30 wt% AgBr/CA block, for instance, weighs only 0.0905 g, corresponding to a density of 15.08 mg·cm^−3^. Unlike conventional powdery photocatalysts, this water-insoluble monolith can be retrieved from the treated water almost without mass loss, a feature highly favourable for repeated practical use [19,20,27].

**Figure 2:**
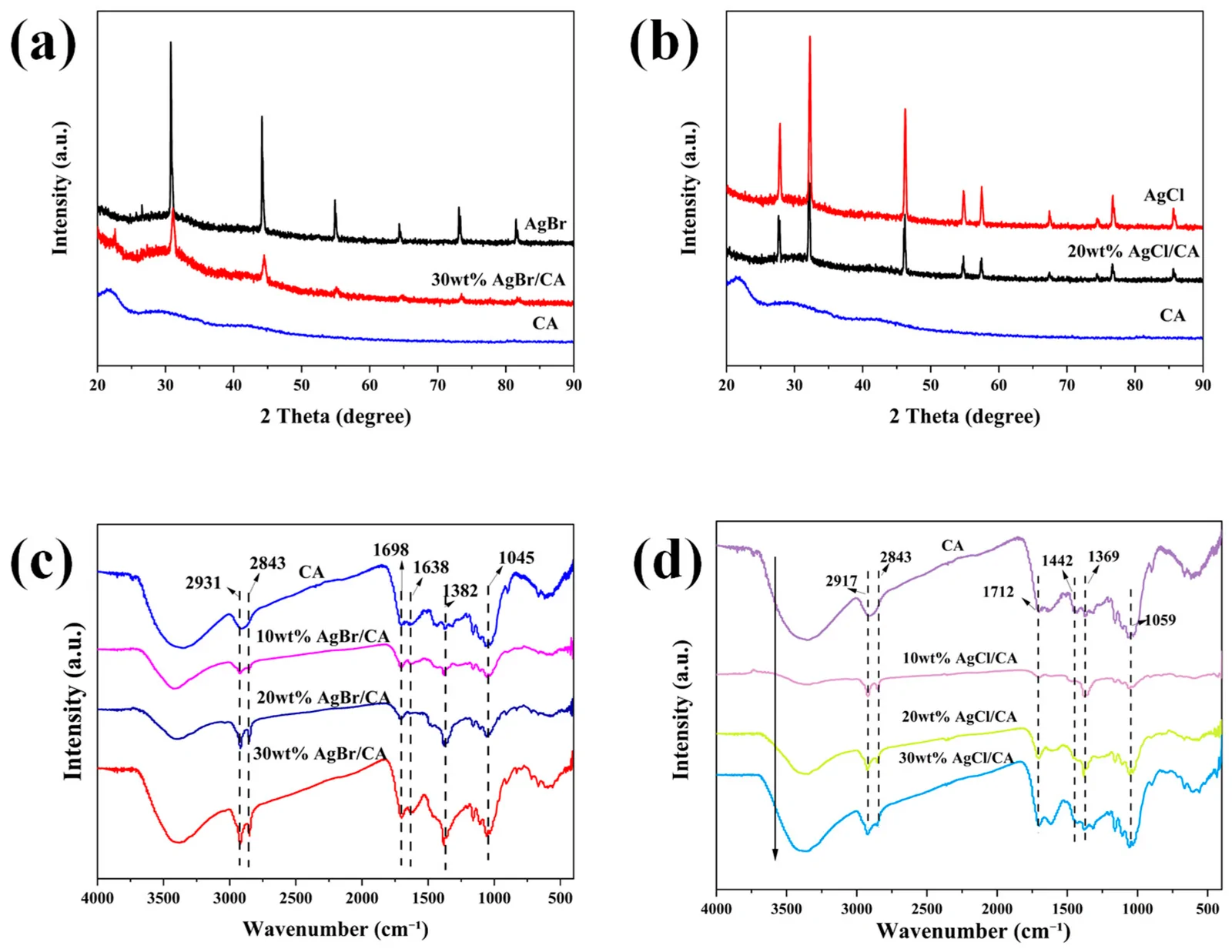
XRD patterns: (**a**) CA, 30 wt% AgBr/CA, and pristine AgBr; (**b**) CA, 20 wt% AgCl/CA, and pristine AgCl. FTIR spectra: (**c**) CA and the 10–30 wt% AgBr/CA composites; (**d**) CA and the 10–30 wt% AgCl/CA composites.

Microscopically, this robust architectural integrity and homogeneous dispersion are strictly dictated by the interfacial chemistry between the carbon skeleton and the AgX nanocrystals. The phase composition and surface chemistry of the composites were examined by XRD and FTIR (Figure 2). The XRD pattern of the bromide system (Figure 2a) shows that pristine CA exhibits a broad hump centered at approximately 22°, characteristic of amorphous carbon containing locally graphitized domains [24]. The 30 wt% AgBr/CA composite displays a series of sharp reflections at 2θ = 30.9°, 44.3°, 55.0°, 64.4°, 73.2°, and 81.6°, indexed to the (200), (220), (222), (400), (420), and (422) planes of face-centered cubic AgBr (JCPDS No. 79-0149) [19]; no diffraction assignable to metallic Ag is detected, confirming that the composite consists solely of the AgBr and carbon phases and that no appreciable reduction of Ag^+^ occurred during synthesis. The chloride system behaves analogously (Figure 2b): besides the carbon hump of CA, the 20 wt% AgCl/CA composite and pristine AgCl exhibit well-defined reflections at 27.8°, 32.2°, 46.2°, 54.8°, 57.5°, 67.5°, 74.5°, 76.7°, and 85.7°, in excellent agreement with the (111), (200), (220), (311), (222), (400), (331), (420), and (422) planes of cubic chlorargyrite AgCl (JCPDS No. 31-1238) [20]. The diffraction intensities of both silver halides increased progressively with the AgX loading from 10 to 30 wt%, while the peak positions remained unchanged, as shown by the full loading series in Figure S1. The coexistence of the characteristic silver-halide reflections with the carbon background, and the absence of any metallic Ag reflection in either system, confirm the successful in situ construction of phase-pure AgBr/CA and AgCl/CA composites without detectable crystalline impurities.

**Figure 3:**
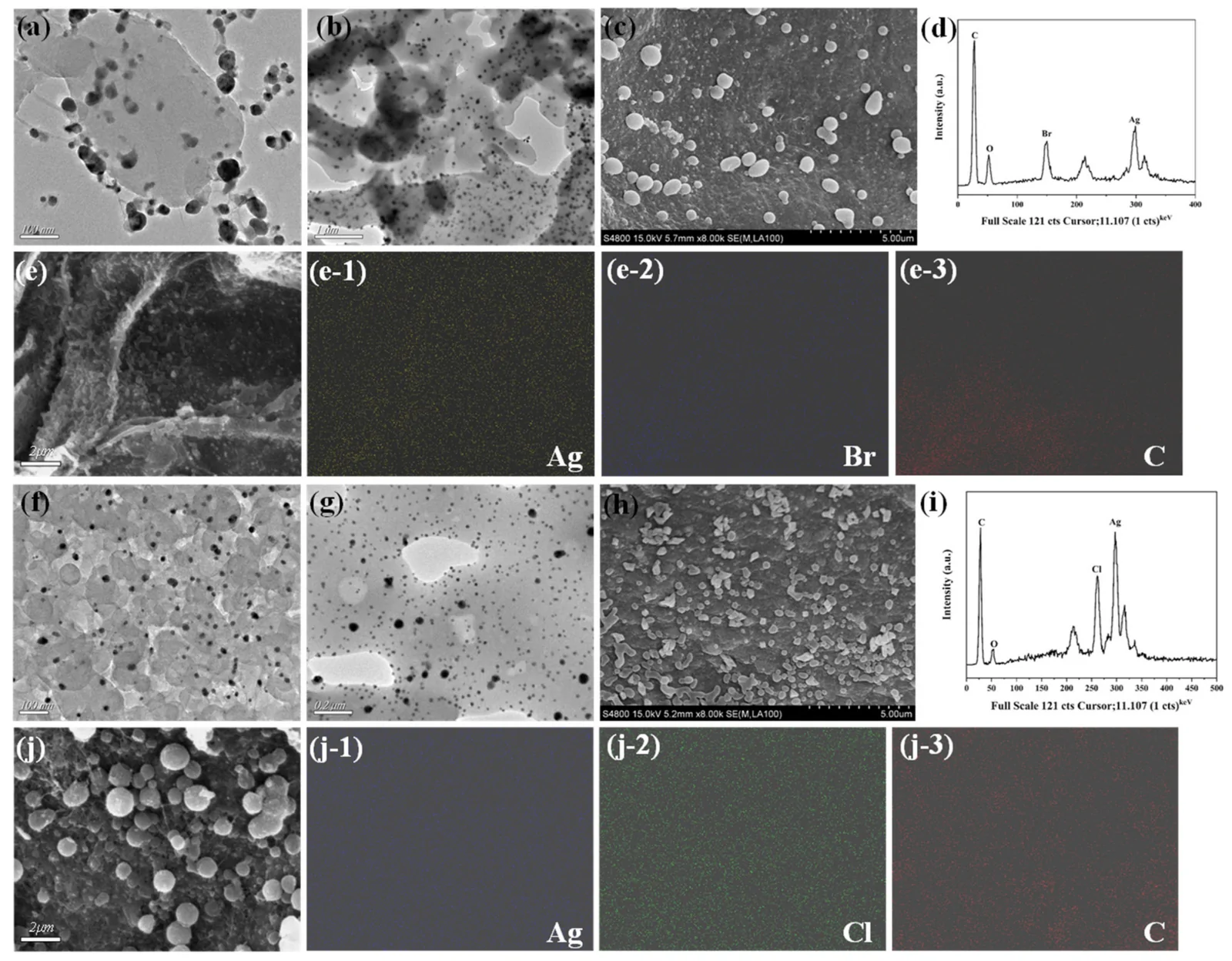
Morphology and elemental distribution of the 30 wt% composites. AgBr/CA: (**a**,**b**) TEM images, (**c**) SEM image, (**d**) EDS spectrum, and (**e**,**e-1**–**e-3**) elemental mapping of Ag, Br, and C. AgCl/CA: (**f**,**g**) TEM images, (**h**) SEM image, (**i**) EDS spectrum, and (**j**,**j-1**–**j-3**) elemental mapping of Ag, Cl, and C.

The corresponding FTIR spectra reveal the surface functional groups of the carbon framework. For the bromide system (Figure 2c), pristine CA exhibits a broad band at approximately 3450 cm^−1^ assigned to O–H stretching of hydroxyl groups and adsorbed water, bands at 2931 and 2843 cm^−1^ arising from asymmetric and symmetric C–H stretching of methylene units, absorptions at 1698 and 1638 cm^−1^ attributed to C=O stretching and aromatic C=C vibrations, and bands at 1382 and 1045 cm^−1^ corresponding to C–H bending and C–O/C–O–C stretching, respectively [24]. The spectra of the 10–30 wt% AgBr/CA composites are essentially superimposable on that of CA, indicating that the introduction of AgBr does not perturb the surface chemistry of the carbon skeleton. A consistent picture is obtained for the chloride system (Figure 2d): CA shows C–H stretching bands at 2917 and 2843 cm^−1^, a C=O stretching band of carboxyl/carbonyl groups at 1712 cm^−1^, C–H bending/carboxylate bands at 1442 and 1369 cm^−1^, and a C–O–C stretching band at 1059 cm^−1^, together with the broad O–H envelope near 3400 cm^−1^; all of these features are retained in the 10–30 wt% AgCl/CA composites [28]. The persistence of these oxygen-containing groups is significant in two respects: (i) they provide the anchoring sites that capture Ag^+^ and direct the uniform in situ nucleation of the AgX nanocrystals—consistent with the homogeneous elemental distributions revealed by the EDS mapping in Figure 3; (ii) oxygen-containing groups may improve the affinity of the carbon surface toward aqueous pollutants, thereby laying the groundwork for the adsorption–photocatalysis synergy discussed below [27,29].

Consequently, the targeted interfacial nucleation translates into an optimal micro-morphology, maximizing the accessible electroactive area for subsequent charge transfer. The microstructure and elemental distribution of the optimal 30 wt% composites were examined by electron microscopy (Figure 3). For AgBr/CA, the TEM images (Figure 3a,b) reveal quasi-spherical AgBr nanoparticles uniformly anchored on the thin carbon sheets. Statistical analysis of more than 100 nanoparticles gives a mean particle diameter of 25.31 ± 9.65 nm, and the corresponding particle-size distribution is presented in Figure S3a. The absence of pronounced particle aggregation demonstrates the effective spatial confinement provided by the carbon framework during the in situ growth process [19]. The SEM image (Figure 3c) further confirms that the AgBr nanoparticles are evenly distributed across the porous carbon framework. The EDS spectrum (Figure 3d) detects C, O, Ag, and Br; the oxygen signal originates from the surface functional groups of the CA. The elemental maps (Figure 3(e-1–e-3)) demonstrate that Ag, Br, and C are homogeneously distributed throughout the selected region, verifying the uniform dispersion of AgBr over the carbon support.

The AgCl/CA composite exhibits a similar microstructure. As shown in Figure 3f,g, numerous AgCl nanoparticles are uniformly distributed over the carbon matrix, with a mean particle diameter of 19.63 ± 3.83 nm based on the statistical analysis of more than 100 particles (Figure S3b). The relatively narrow particle-size distribution further confirms that the carbon framework effectively restricts nanoparticle growth and aggregation, and the SEM image (Figure 3h) confirms their even distribution over the interconnected porous network. The EDS spectrum (Figure 3i) identifies C, O, Ag, and Cl, and the corresponding maps (Figure 3(j-1–j-3)) reveal the homogeneous spatial distribution of Ag, Cl, and C. In both systems, the intimate and uniform AgX–carbon interfacial contact maximizes the area available for interfacial charge transfer and restrains nanoparticle growth and aggregation, providing the structural foundation for the enhanced photocatalytic activity and stability discussed in the following sections [24].

### 2.2. Contrasting Optical Modulation and Surface Electronic States

**Figure 4:**
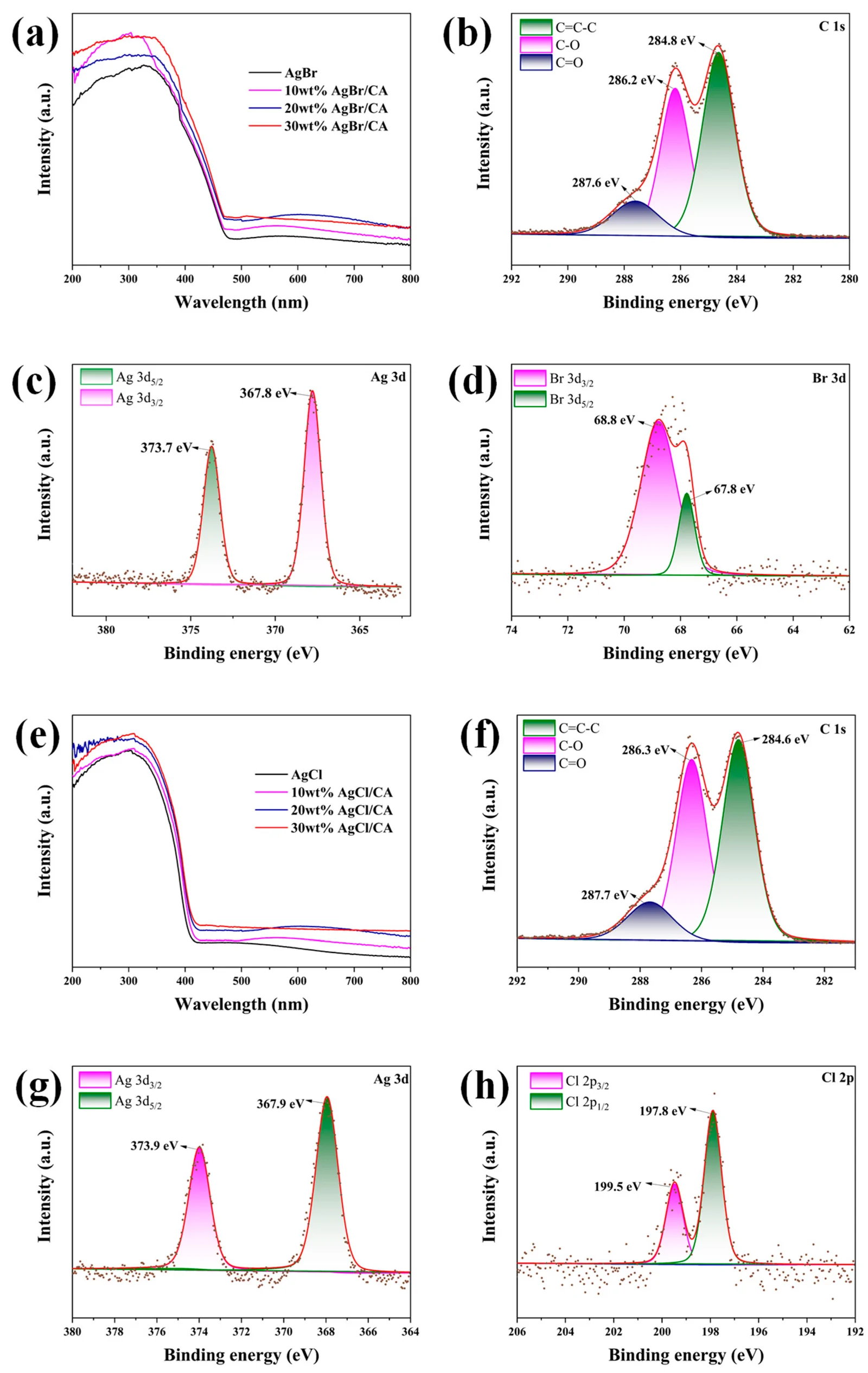
Optical and surface-electronic characterization of the AgX/CA composites. AgBr system: (**a**) UV–vis DRS of AgBr and the 10–30 wt% AgBr/CA composites, and high-resolution XPS spectra of (**b**) C 1s, (**c**) Ag 3d, and (**d**) Br 3d of 30 wt% AgBr/CA. AgCl system: (**e**) UV–vis DRS of AgCl and the 10–30 wt% AgCl/CA composites, and high-resolution XPS spectra of (**f**) C 1s, (**g**) Ag 3d, and (**h**) Cl 2p of 30 wt% AgCl/CA.

The integration of the 3D carbon aerogel reshapes the light-harvesting kinetics, playing a contrasting yet universally beneficial optical role for both halide systems. The light-harvesting capability and surface electronic states of the composites were investigated by UV–vis diffuse reflectance spectroscopy (DRS) and XPS (Figure 4). The DRS spectrum of pristine AgBr shows an absorption edge extending into the visible-light region. Based on the direct-allowed Tauc plot shown in Figure S4a, the optical band gap of pristine AgBr was estimated to be approximately 2.82 eV [19]. Upon coupling with CA, the AgBr/CA composites exhibit markedly intensified absorption across the entire visible region, which is ascribed to the broadband absorption of the black carbonaceous skeleton superimposed on the intrinsic AgBr absorption, and the absorbance increases progressively with the carbon-relative loading. In contrast, pristine AgCl exhibits a relatively sharp absorption edge near 400 nm. The corresponding direct-allowed Tauc plot gives an estimated optical band gap of approximately 3.04 eV for pristine AgCl (Figure S4b), indicating that bare AgCl has only limited absorption in the visible-light region [14]. The optical band-gap values were determined by extrapolating the linear portions of the [αhν]^2^-versus-hν plots to the photon-energy axis, assuming direct allowed transitions. In contrast, all AgCl/CA composites display substantially enhanced absorbance throughout the 400–800 nm range relative to bare AgCl. This comparison highlights the distinct yet complementary roles of the carbon aerogel in the two systems: whereas the CA merely amplifies the pre-existing visible-light response of AgBr, it endows the otherwise UV-confined AgCl with visible-light-harvesting ability, thereby establishing the very prerequisite for its visible-light photocatalysis. The intrinsic band gaps of the two halides originate from the hybridization between the Ag^+^ 4d levels and the halide p states [14]; consequently, the markedly different visible-light behavior observed here is governed primarily by the carbon-induced absorption rather than by the silver-halide host itself.

XPS was performed to determine the surface chemical states of the two composites. As shown in Figure S5a, the survey spectrum of 30 wt% AgBr/CA contains the characteristic signals of C, O, Ag, and Br, whereas the spectrum of 30 wt% AgCl/CA displays the corresponding C, O, Ag, and Cl signals (Figure S5b). Quantitative analysis of the survey spectra shows that the atomic percentages of C, O, Ag, and Br in 30 wt% AgBr/CA are 68.37%, 29.63%, 0.86%, and 1.05%, respectively, while those of C, O, Ag, and Cl in 30 wt% AgCl/CA are 67.99%, 30.64%, 0.75%, and 0.62%, respectively (Table S1). The predominance of C and O is consistent with the carbon-rich, partially oxygenated aerogel framework, whereas the Ag and halide signals verify the successful surface incorporation of the silver-halide nanoparticles.

High-resolution XPS spectra were subsequently analyzed to determine the chemical states of the constituent elements. For 30 wt% AgBr/CA, the C 1s spectrum (Figure 4b) is deconvoluted into three components at 284.8, 286.2, and 287.6 eV, assigned to C–C/C=C, C–O, and C=O species of the partially oxygenated carbon skeleton [30]. The Ag 3d spectrum (Figure 4c) shows the Ag 3d_5/2_ and Ag 3d_3/2_ peaks at 367.8 and 373.7 eV with a spin–orbit splitting of 5.9 eV, characteristic of Ag^+^ in AgBr, and the Br 3d doublet (Figure 4d) appears at 67.8 (3d_5/2_) and 68.8 eV (3d_3/2_), corresponding to lattice Br^−^ [31,32,33]. The chloride composite exhibits an entirely consistent picture: the C 1s spectrum (Figure 4f) resolves into C–C/C=C (284.6 eV), C–O (286.3 eV), and C=O (287.7 eV) components; the Ag 3d peaks (Figure 4g) at 367.9 and 373.9 eV (splitting 6.0 eV) confirm Ag^+^ in AgCl; and the Cl 2p doublet (Figure 4h) at 197.8 (2p_3/2_) and 199.5 eV (2p_1/2_) is ascribed to lattice Cl^−^ [34,35]. In both systems, the Ag 3d binding energies and spin–orbit splitting are diagnostic of Ag^+^ rather than metallic Ag^0^, in agreement with the XRD results and confirming that the silver species remain in the +1 oxidation state after the in situ co-precipitation and freeze-drying process.

### 2.3. Multi-Class Photocatalytic Performance and Macroscopic Recoverability

To quantitatively evaluate the hierarchical porosity, N_2_ adsorption–desorption measurements were performed (Figure S7). Pristine CA exhibits a relatively low specific surface area of 10.23 m^2^/g, primarily characterized by micropores. Markedly, upon the in situ growth of the AgX nanoparticles, the specific surface areas of the 30 wt% AgBr/CA and 30 wt% AgCl/CA composites significantly increased to 45.67 and 62.31 m^2^/g, respectively. This enhancement demonstrates that the densely anchored nanocrystals act as effective nano-spacers, preventing the severe restacking of the carbon skeleton during the freeze-drying process. Furthermore, the composites evolve into pronounced mesoporous architectures (with dominant pore sizes of 20–40 nm and 10–20 nm, respectively) and present expanded total pore volumes (~0.26 and ~0.30 cm^3^/g). This optimized hierarchical mesoporous network intrinsically facilitates the rapid mass transfer and deep pre-concentration of pollutant molecules near the photoactive sites, corroborating the strong dark-adsorption capabilities observed. Compared to conventional powdery AgX photocatalysts, which typically suffer from severe self-agglomeration and exhibit extremely low surface areas (often <5 m^2^/g), the significantly expanded surface areas of our monolithic composites directly demonstrate the structural superiority of the 3D aerogel architecture in exposing abundant micro-interfacial active sites.

**Figure 5:**
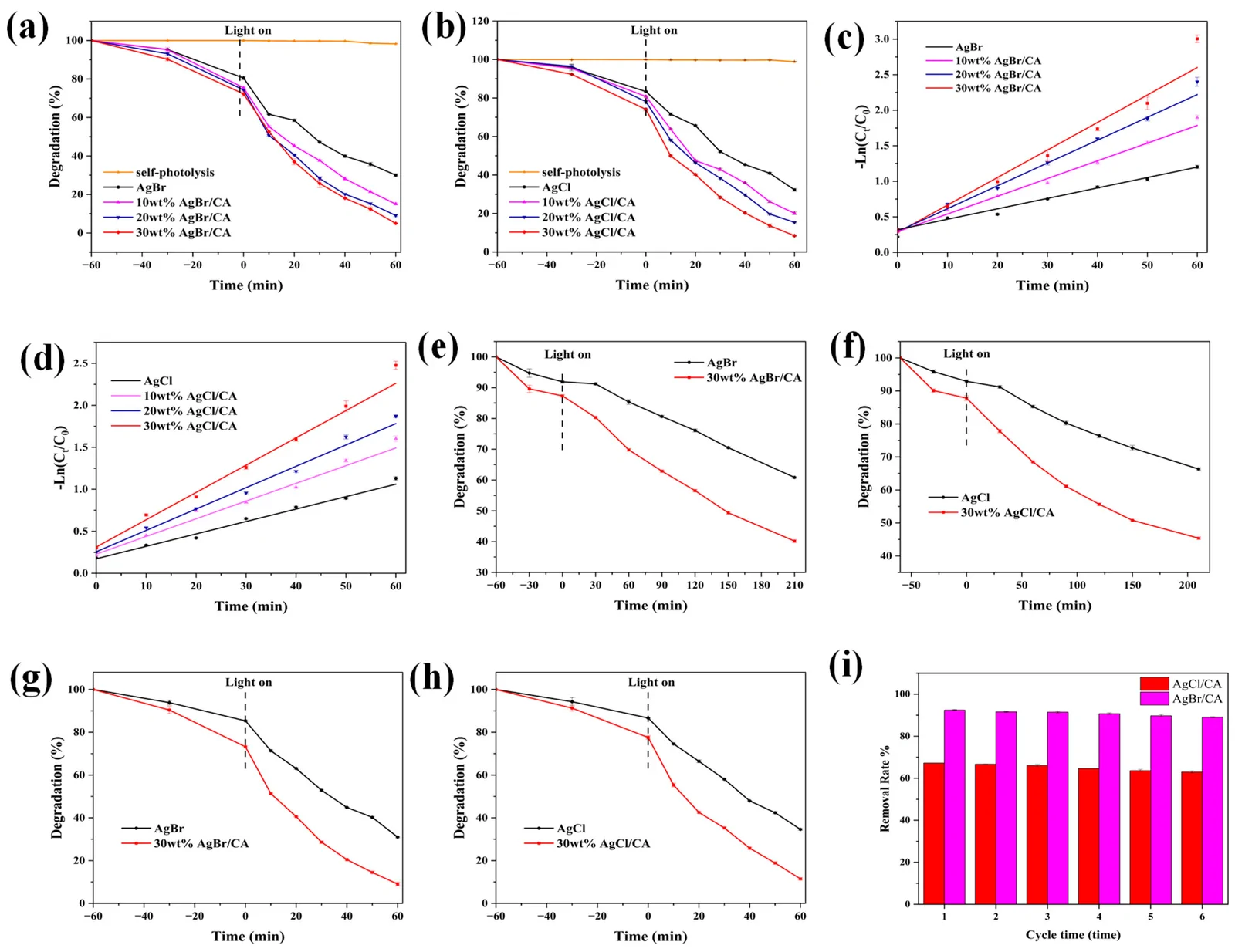
Photocatalytic performance of the AgX/CA composites under Xe-lamp irradiation. MB degradation curves over the (**a**) AgBr and (**b**) AgCl systems, and the corresponding pseudo-first-order kinetics for the (**c**) AgBr and (**d**) AgCl systems; degradation of CIP over the (**e**) AgBr and (**f**) AgCl systems; degradation of RhB over the (**g**) AgBr and (**h**) AgCl systems; and (**i**) removal efficiency of 30 wt% AgBr/CA and 30 wt% AgCl/CA over six consecutive RhB degradation cycles.

To enable a direct and controlled comparison among the synthesized photocatalysts, all samples were evaluated under a unified protocol using the same pollutant concentration (10 mg L^−1^), solution volume (40 mL), catalyst dosage (40 mg), solution conditions, light source, and irradiation procedure. No sample-specific pH adjustment was applied. The micro-interfacial anchoring and optical amplification directly translate into exceptional, rapid photocatalytic mineralization. The photocatalytic activity of the two composite systems was evaluated by degrading two cationic dyes (MB and RhB) and a fluoroquinolone antibiotic (CIP) under visible-light irradiation (λ ≥ 420 nm) from a 350 W Xe lamp, using 40 mg of catalyst in 40 mL of a 10 mg·L^−1^ pollutant solution. Prior to illumination, each suspension was stirred in the dark for 60 min to establish adsorption–desorption equilibrium; the pronounced dark-stage uptake by the composites (Figure 5a,b) reflects the pollutant-enrichment capability of the porous, functional-group-rich carbon skeleton [27,29]. A blank test confirmed that the self-photolysis of the dyes was negligible.

As shown in Figure 5a, all AgBr/CA composites degraded MB markedly faster than pristine AgBr under visible light. The activity increased monotonically with AgBr loading over the investigated range (10–30 wt%): after 60 min, 30 wt% AgBr/CA removed 95.68% of MB, compared with only 30.57% for pristine AgBr. The chloride system followed the same trend (Figure 5b), with the degradation efficiency rising in the order AgCl < 10 wt% < 20 wt% < 30 wt% AgCl/CA. The degradation kinetics were analyzed using the pseudo-first-order model, ln(C_0_/C_t_) = kt, and the −ln(C_t_/C_0_) versus t plots were well fit by straight lines for both systems (Figure 5c,d; R^2^ > 0.97, Table 1). The apparent rate constants of AgBr, 10, 20, and 30 wt% AgBr/CA were 0.0147, 0.0249, 0.0321, and 0.0387 min^−1^, respectively, the optimal composite thus exhibiting a rate constant about 2.6 times that of pristine AgBr; for the chloride system, the constants increased from 0.0148 min^−1^ for AgCl to 0.0325 min^−1^ for 30 wt% AgCl/CA, a 2.2-fold enhancement. It is noteworthy that pristine AgCl, despite its wide band gap, exhibited non-negligible MB removal, which can be partly attributed to a dye-sensitization pathway in which photoexcited dye molecules inject electrons into the AgCl conduction band [36]; the genuine photocatalytic origin of the composite activity is corroborated by the degradation of the colorless antibiotic CIP discussed below [37,38].

To unequivocally validate the intrinsic semiconductor-driven mechanism and eliminate the ambiguity of dye-sensitization artifacts, the composites were challenged with diverse recalcitrant pollutants. To assess the generality of the composites, RhB and CIP were tested under identical conditions (Figure 5e–h). For RhB (Figure 5g,h), the optimal 30 wt% AgBr/CA and 30 wt% AgCl/CA composites achieved degradation efficiencies of approximately 91% and 88% within 60 min, substantially exceeding those of pristine AgBr (~69%) and AgCl (~65%). The degradation of CIP proceeded more slowly, as expected from its refractory quinolone skeleton and the absence of any dye-sensitization contribution (Figure 5e,f); nevertheless, within 210 min, 30 wt% AgBr/CA and 30 wt% AgCl/CA removed approximately 60% and 54% of CIP, against only ~39% and ~34% over the corresponding pristine halides. Because CIP is essentially colorless under the irradiation conditions, its degradation cannot be mediated by dye sensitization and therefore directly evidences the semiconductor-based photocatalysis of the composites [37]. Across all three pollutants, the AgBr-based system consistently outperformed its AgCl counterpart by a modest margin, in line with the intrinsic visible-light absorption of AgBr (Figure 4a); the strong activity of AgCl/CA is nonetheless particularly significant, since bare AgCl harvests little visible light, underscoring the decisive contribution of the CA framework to light absorption and charge separation in the chloride system [13,39,40]. To position these results against the current literature, Table 2 compares the photocatalytic performance of the present AgX/CA composites with representative AgX-based photocatalysts reported recently. The degradation efficiency of our composites is competitive with, and in several cases exceeds, that of these benchmark systems under comparable conditions. More importantly, whereas nearly all benchmark systems are deployed as powders or fragile foams requiring centrifugation or filtration for recovery, the present monolithic composites can be retrieved directly and near-losslessly from the treated water. This combination of competitive activity, a renewable and low-cost biomass precursor, and facile macroscopic recovery distinguishes the monolithic aerogel architecture from prior AgX photocatalysts and underscores its practical relevance for real-world water treatment.

As summarized in Table 2, the photocatalytic performance of the present AgX/CA composites is competitive with that of recently reported AgX-based heterojunction photocatalysts. Direct comparison of degradation efficiencies should be approached cautiously because the reported studies differ substantially in pollutant type and concentration, catalyst dosage, irradiation wavelength, and reaction time. For example, the catalyst concentrations used in the reference systems range from 0.2 to 1.0 g L^−1^, while the irradiation times range from 60 to 180 min. Furthermore, the kinetic constants cannot always be compared directly because different kinetic models and units were adopted. Nevertheless, the present AgX/CA composites combine competitive visible-light photocatalytic activity with a free-standing monolithic architecture, enabling direct macroscopic recovery without the centrifugation or filtration generally required for dispersed powder photocatalysts.

Ultimately, the free-standing architecture functionally resolves the persistent operational bottlenecks of powdered photocatalysts. Over six consecutive cycling evaluations (Figure 5i), the monolithic composites retain exceptional catalytic robustness with only marginal efficiency fluctuations. After each cycle, the monolithic catalyst was retrieved, washed, and reused. The removal efficiency of 30 wt% AgBr/CA declined only marginally from ~93% to ~89% over six cycles, and 30 wt% AgCl/CA likewise retained ~63% of its initial ~67%, corresponding to losses of merely a few percentage points. Such limited decay—together with the near-lossless recovery afforded by the free-standing monolith—demonstrates the good operational durability of both composites and stands in contrast to the rapid deactivation typically suffered by bare silver halides through the photoreduction of lattice Ag^+^ to metallic Ag^0^ [17,20,41].

To fundamentally validate the anti-photocorrosion capability and long-term durability of the composites, the post-reaction structural and chemical integrity of the retrieved catalysts was examined after six consecutive cycles (Figure S8). Crucially, the post-cycling XRD patterns confirm the absolute absence of metallic Ag^0^ diffraction peaks, explicitly validating that the 3D “electron reservoir” architecture effectively and rapidly extracts photogenerated electrons, thereby fundamentally starving the intrinsic self-reduction pathway of AgX. Moreover, the post-reaction SEM images reveal that the interconnected macroporous carbon framework and the homogeneous nanoparticle dispersion are robustly maintained without severe aggregation or structural collapse, well rationalizing the excellent macroscopic cycling stability.

It is worth noting that while natural biomass precursors typically exhibit inherent batch-to-batch variability in tissue structure, the consistently small standard deviations across our independent triplicate evaluations (Figure 5) indicate that the standardized hydrothermal and freeze-drying protocol effectively ensures robust laboratory-scale reproducibility. Nevertheless, addressing broader seasonal or regional variations in the precursor’s porosity and composition remains a critical consideration for the future large-scale industrial deployment of these monoliths.

### 2.4. Photocatalytic Mechanisms and Charge-Carrier Dynamics

**Figure 6:**
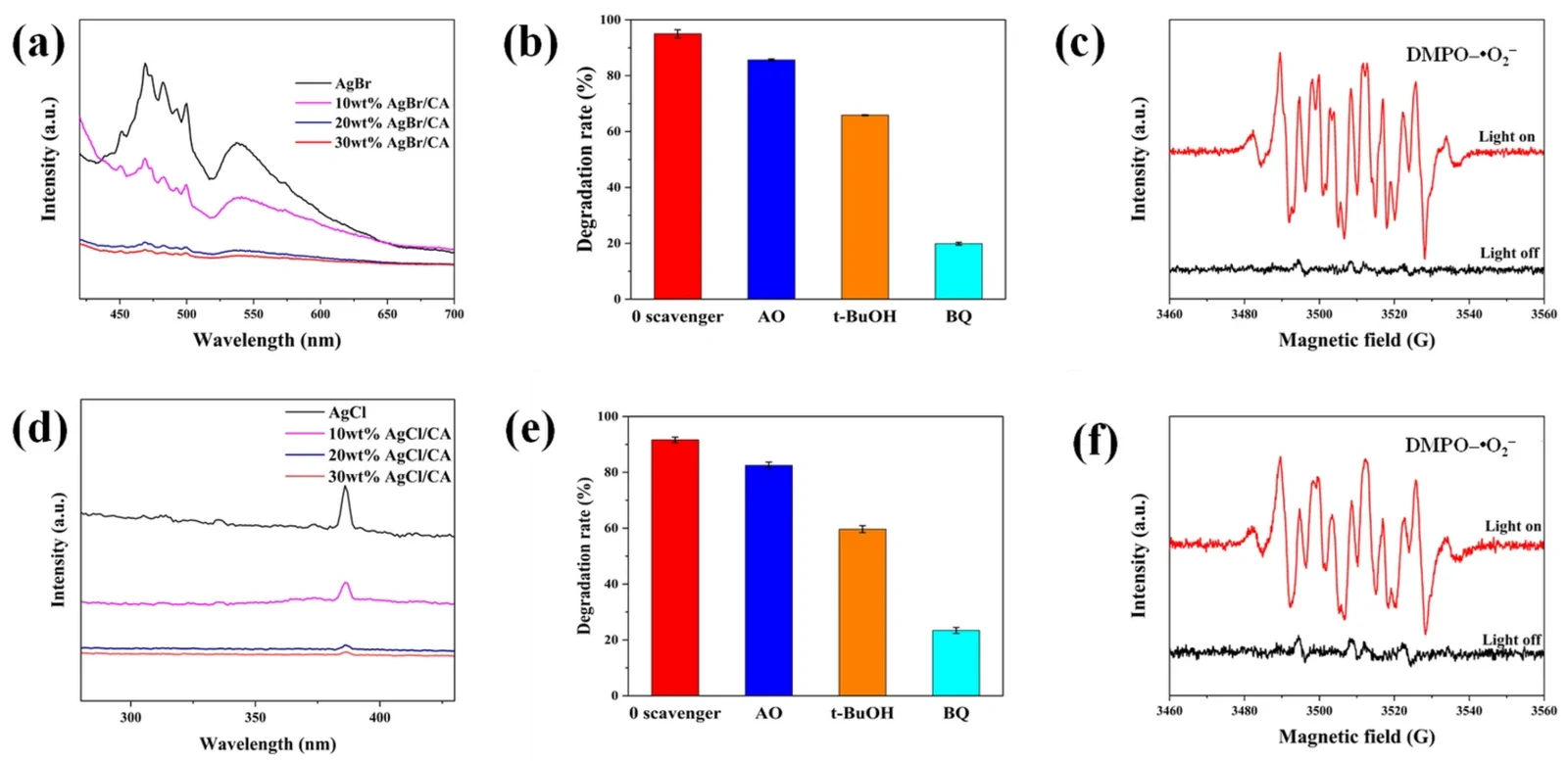
Mechanistic characterization of the AgX/CA composites. AgBr system: (**a**) PL spectra of pristine AgBr and the 10–30 wt% AgBr/CA composites; (**b**) effects of radical scavengers on MB degradation over 30 wt% AgBr/CA; (**c**) DMPO–•O_2_^−^ ESR spectra of 30 wt% AgBr/CA in the dark and under visible-light irradiation. AgCl system: (**d**) PL spectra of pristine AgCl and the 10–30 wt% AgCl/CA composites; (**e**) effects of radical scavengers on MB degradation over 30 wt% AgCl/CA; (**f**) DMPO–•O_2_^−^ ESR spectra of 30 wt% AgCl/CA in the dark and under visible-light irradiation.

To elucidate the origin of the enhanced activity, the charge-carrier behavior and reactive species of the composites were probed by PL spectroscopy, radical-trapping experiments, and ESR (Figure 6). Steady-state PL spectroscopy was used to examine the radiative recombination behavior of the photogenerated charge carriers. As shown in Figure 6a,d, the AgBr/CA and AgCl/CA composites exhibit lower PL emission intensities than pristine AgBr and AgCl, respectively. This PL quenching is consistent with suppressed radiative recombination and facilitated charge separation after interfacial coupling with the carbon aerogel.

To further unravel the ultrafast charge-carrier dynamics, time-resolved photoluminescence (TRPL) decay spectra were recorded (Figure S6). The average emission lifetime of the optimal 30 wt% AgBr/CA composite is prolonged to 0.75 ns compared to pristine AgBr (0.69 ns). An analogous prolongation is observed for the chloride system (from 0.72 ns for AgCl to 0.85 ns for 30 wt% AgCl/CA). This dynamically prolonged lifetime directly corroborates that the photogenerated electrons are efficiently extracted and delocalized by the continuous carbon aerogel network, successfully delaying the charge recombination process and matching the proposed electron-reservoir mechanism. However, because steady-state PL measurements do not directly quantify interfacial charge-transfer kinetics, these results are interpreted together with the radical-trapping and ESR measurements discussed below.

The reactive species were identified by radical-trapping experiments, using ammonium oxalate (AO), tert-butanol (t-BuOH), and benzoquinone (BQ) as scavengers for holes (h^+^), hydroxyl radicals (•OH), and superoxide radicals (•O_2_^−^), respectively. For 30 wt% AgBr/CA (Figure 6b), the addition of AO caused only a slight decline in MB degradation, t-BuOH led to a moderate suppression, and BQ depressed the efficiency most strongly (from 95.68% to about 20%). An entirely analogous hierarchy was observed for 30 wt% AgCl/CA (Figure 6e). These results consistently indicate that •O_2_^−^ is the dominant reactive species in both systems, •OH plays a secondary role, and direct hole oxidation contributes only marginally. ESR spin-trapping measurements with DMPO corroborate this assignment: characteristic signals of the DMPO–•O_2_^−^ adduct emerged under illumination for both 30 wt% AgBr/CA (Figure 6c) and 30 wt% AgCl/CA (Figure 6f) while remaining negligible in the dark, providing direct evidence for the photoinduced generation of superoxide radicals [39,42,43].

The combination of a marginal hole-scavenging effect with an appreciable •OH contribution suggests that •OH is generated mainly through an electron-mediated pathway rather than by direct hole oxidation of water. As established for analogous AgX/aerogel systems, the electrons accumulated on the carbon network are first scavenged by dissolved O_2_ to form •O_2_^−^ via one-electron reduction (O_2_ + e^−^ → •O_2_^−^), part of which is subsequently converted into •OH through a stepwise reduction route involving H_2_O_2_ (O_2_ + 2H^+^ + 2e^−^ → H_2_O_2_ → •OH) [19,43]. The photoinduced formation of •OH over AgBr/CA was further confirmed by the DMPO–•OH ESR signal that emerged only under illumination (Figure S2). This electron-driven scenario is fully consistent with the strong PL quenching, since the efficient extraction of electrons by the CA framework simultaneously feeds the O_2_-reduction chain and depletes the recombination channel.

On the basis of these results, a unified mechanism for the AgX/CA composites can be proposed. Under irradiation, the AgX component is photoexcited to generate electron–hole pairs; owing to the intimate interfacial contact established by the in situ growth, the photogenerated electrons migrate rapidly from the AgX conduction band into the carbon aerogel, where they reduce dissolved O_2_ to •O_2_^−^ (assisted by the further formation of •OH), while the holes left in the AgX valence band contribute to a lesser extent. The reactive species, predominantly •O_2_^−^ aided by •OH, then attack and mineralize the adsorbed pollutant molecules. Beyond promoting charge separation, the CA framework contributes through two additional channels: its porous, functional-group-rich surface pre-concentrates pollutants near the active AgX sites, shortening the diffusion path of the short-lived radicals [17,20]. This proposed electron-diversion effect is consistent with the good cycling stability observed in Figure 5i. Notably, unlike Ag/AgX plasmonic photocatalysts whose activity relies on the in situ generation of metallic Ag^0^, the present composites contain no detectable Ag^0^ after synthesis (XRD and XPS, Figure 2 and Figure 4), indicating that their as-prepared activity does not depend on photocorrosion-derived metallic silver [14,18].

The two halide systems share this common framework but differ in the photoexcitation step: for AgBr/CA, the intrinsically visible-light-active AgBr is directly excited, and the CA further amplifies light harvesting; meanwhile, for AgCl/CA—whose AgX component absorbs little visible light—the photoresponse relies primarily on the composite-induced visible absorption (Figure 4e), which rationalizes both its substantial composite activity and its slightly lower performance relative to the bromide counterpart.

Taken together, the characterization data trace a coherent structure–electronic–activity relationship for both halide systems. Structurally, in situ surfactant-directed nucleation produces intimate AgX–carbon interfacial contact and homogeneous nanoparticle dispersion (Figure 3), which maximizes the interfacial area available for charge transfer. This interfacial intimacy is the prerequisite for the optical and electronic consequences observed downstream: the black carbon skeleton intensifies visible-light harvesting (Figure 4a,e), while the lower PL emission intensities of the AgX/CA composites relative to the pristine silver halides are consistent with suppressed radiative recombination after interfacial coupling with the carbon framework (Figure 6a,d). These electronic effects translate directly into function: the apparent first-order rate constant rises monotonically with loading (Table 1) in lockstep with the deepening PL quenching, and the trapping and ESR results (Figure 6b,c,e,f) further support enhanced •O_2_^−^ generation in the AgX/CA composites. The consistency of this trend across the AgBr and AgCl systems—differing only in the magnitude set by their intrinsic visible absorption—indicates that the carbon scaffold governs performance through a unified charge-separation mechanism rather than through halide-specific effects.

**Figure 7:**
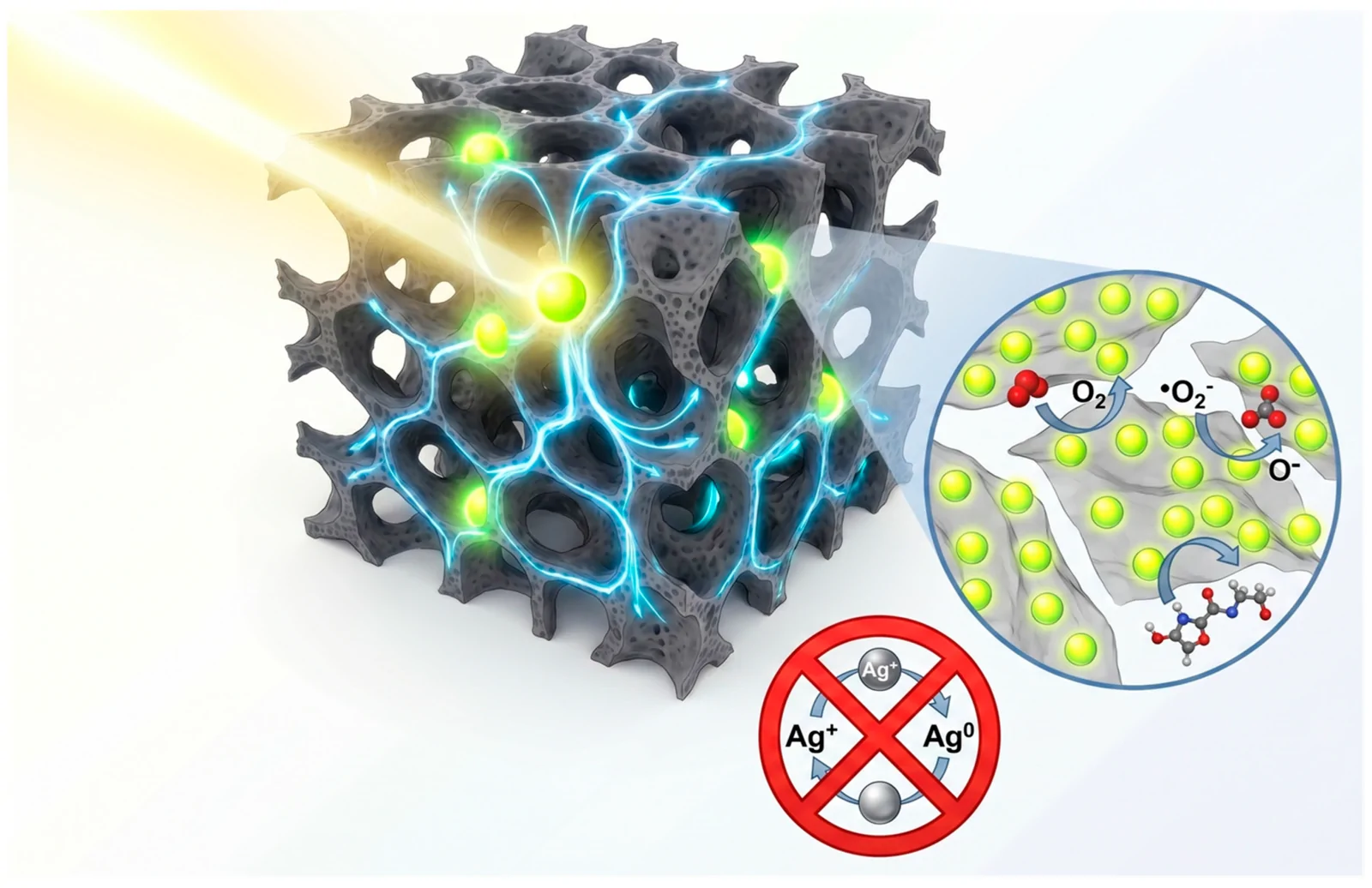
Comprehensive schematic illustration of the photocatalytic mechanism over the free-standing AgX/CA composites.

To provide a comprehensive and intuitive overview of the overall photocatalytic process, the proposed mechanism is systematically summarized in Figure 7. Upon visible-light irradiation, the 3D carbon aerogel framework functioned as an ultrafast electron reservoir to extract and delocalize the photogenerated electrons from the conduction band of the anchored AgX nanocrystals (charge transfer pathways). This directed electron extraction fundamentally suppresses the intrinsic photocorrosion of AgX (i.e., preventing the self-reduction of Ag^+^ to Ag^0^) while simultaneously promoting the continuous reduction of dissolved O_2_ into massive superoxide (⋅O_2_^−^) and hydroxyl (⋅OH) radicals (reactive oxygen species generation). Driven by the hierarchical porosity, the target molecules are locally enriched near these active micro-interfaces, where they are incessantly attacked and mineralized by the abundantly generated ROS (pollutant degradation steps). This synergistic structure–electronic–activity relationship seamlessly unifies our macroscopic kinetics and in situ spectroscopic observations (PL, trapping, and ESR) into a coherent mechanistic framework.

## 3. Conclusions

In summary, we have developed a monolithic AgX/biomass carbon aerogel (CA) composite platform that addresses the coupled activity–recoverability challenge in silver-halide photocatalysis. By intimately anchoring AgX nanocrystals in situ onto a 3D hierarchical carbon skeleton, the CA network functions as an ultrafast electron reservoir while playing contrasting optical roles—amplifying the intrinsic visible-light absorption of AgBr and endowing AgCl with unprecedented visible-light responsiveness. Consequently, the composites deliver exceptional, multi-class mineralization capabilities against both dyes and colorless antibiotics, alongside robust macroscopic structural stability over consecutive cycles. Mechanistic investigations indicate that coupling with the carbon framework facilitates charge separation and promotes superoxide-radical generation, thereby enhancing photocatalytic activity. Ultimately, this work mitigates the key microscale and macroscale limitations of conventional powdery photocatalysts and establishes a scalable, sustainable architectural paradigm for designing highly efficient and practically recoverable water-remediation systems.

While this fundamental study successfully establishes a scalable proof-of-concept for the monolithic “electron reservoir” architecture, we thoughtfully acknowledge certain laboratory-scale limitations. Specifically, the small batch-volume limits of the current testing system restricted the continuous extraction of large-volume aliquots required for quantitative total organic carbon (TOC) mineralization profiling and high-resolution LC-MS intermediate tracking. Furthermore, the absolute quantification of trace-metal leaching (e.g., via ICP-MS) and the relatively energy-intensive nature of the current freeze-drying step represent practical constraints that warrant further methodological refinement.

To explicitly bridge the gap between this fundamental proof-of-concept and practical industrial implementation, our future research will prioritize several critical directions. First, systematic screening across diverse biomass precursors will be conducted to further optimize the precursor-dependent structure–activity relationships. Second, ultra-long-term continuous-flow stability evaluations will be implemented to comprehensively validate the ultimate operational durability of the monoliths. Finally, transitioning toward low-energy ambient-pressure drying technologies, coupled with comprehensive pilot-scale Techno-Economic Assessments (TEAs), will serve as the crucial next milestone to maximize the life-cycle sustainability and commercial viability of these macroscopic environmental catalysts.

## 4. Materials and Methods

### 4.1. Materials

Cetyltrimethylammonium bromide (CTAB), cetyltrimethylammonium chloride (CTAC), silver nitrate (AgNO_3_), ethanol, methylene blue (MB), rhodamine B (RhB), and ciprofloxacin (CIP) were of analytical grade and supplied by Sinopharm Chemical Reagent Co., Ltd. (Shanghai, China). Ammonium oxalate (AO), tert-butanol (t-BuOH), and p-benzoquinone (BQ) were used as the radical scavengers, and 5,5-dimethyl-1-pyrroline-N-oxide (DMPO) was used as the spin-trapping agent; all were also obtained from Sinopharm Chemical Reagent Co., Ltd. (Shanghai, China). Fresh winter melon was purchased from a local market. Deionized water was made from laboratory water purification equipment.

### 4.2. Synthesis of the Biomass Carbon Aerogel (CA)

Fresh winter melon was peeled, and the soft flesh was cut into cubes of approximately 2 cm × 2 cm × 4 cm. After being rinsed with distilled water for 10 min, the cubes were sealed in a Teflon-lined stainless-steel autoclave and hydrothermally treated at 180 °C for 12 h. After cooling naturally to room temperature, the product was washed repeatedly with a water/ethanol mixture (1:1, *v*/*v*) to remove soluble impurities, affording a carbonaceous wet-gel. Direct freeze-drying of this wet-gel yielded the pristine CA used for reference characterization.

### 4.3. Synthesis of the AgBr/CA Composites

A given amount of the carbonaceous wet-gel was immersed in 20 mL of a CTAB water/ethanol mixed solution (4:1, *v*/*v*) for 12 h under magnetic stirring at room temperature, during which the cationic surfactant adsorbed onto the carbon surface and served as the bromide source. An AgNO_3_ aqueous solution (20 mL, 0.02 mol·L^−1^) was then prepared, and the CTAB-impregnated wet-gel was immersed in it for a further 12 h under magnetic stirring at room temperature, allowing AgBr to nucleate in situ on the gel skeleton. After the in situ precipitation step, the obtained AgBr/CA wet gels were washed three times with deionized water to effectively remove the highly soluble nitrate residues and unreacted surfactants and subsequently freeze-dried (FD-1C-50 freeze dryer, Boyikang, Beijing, China) to afford the AgBr/CA composite. By varying the amounts of CTAB and AgNO_3_ while keeping the carbon aerogel fixed, composites with nominal AgBr mass fractions of 10, 20, and 30 wt% were prepared (denoted as x wt% AgBr/CA). For comparison, pristine AgBr was synthesized through the same procedure without the carbon aerogel.

### 4.4. Synthesis of the AgCl/CA Composites

The obtained AgCl/CA wet gels were washed several times with deionized water and subsequently freeze-dried using the same procedure, except that CTAC was used in place of CTAB as both the surface-directing agent and the chloride source. By analogously varying the amounts of CTAC and AgNO_3_, composites with nominal AgCl mass fractions of 10, 20, and 30 wt% were obtained (denoted as x wt% AgCl/CA), and pristine AgCl was prepared in the same manner without the carbon aerogel.

### 4.5. Material Characterizations

X-ray diffraction (XRD) patterns were recorded on a D8 Advance diffractometer (Bruker AXS, Karlsruhe, Germany) with Cu Kα radiation over a 2θ range of 10–90°. Fourier-transform infrared (FTIR) spectra were collected on a Nicolet Nexus 470 spectrometer (Thermo Electron, Waltham, MA, USA) using the KBr-pellet technique over 400–4000 cm^−1^ at room temperature. The morphology was examined by field-emission scanning electron microscopy (FE-SEM, S-4800, Hitachi, Tokyo, Japan) and transmission electron microscopy (TEM, JEM-2010, JEOL, Tokyo, Japan, 200 kV), and the elemental composition and distribution were analyzed by energy-dispersive X-ray spectroscopy (EDS). X-ray photoelectron spectroscopy (XPS) was performed on a Thermo ESCALAB 250Xi spectrometer. The high-resolution XPS spectra were processed and peak-fitted using Thermo Scientific Avantage software 6.9 with the Powell fitting algorithm. All binding energies were calibrated against the C 1s reference peak at 284.8 eV. UV–vis diffuse reflectance spectra (DRS) were acquired on a UV-2450 spectrophotometer (Shimadzu, Kyoto, Japan) with BaSO_4_ as the reference. Photoluminescence (PL) spectra were measured on a fluorescence spectrometer (Photon Technology International, Monmouth Junction, NJ, USA) that can emit a fluorescence signal with excitation light at 320 nm. Both the excitation and emission slit widths were set to 5.0 nm to ensure optimal signal-to-noise ratios. Electron spin resonance (ESR) spectra were recorded on a Bruker A300 spectrometer(Bruker, Karlsruhe, Germany) using DMPO as the spin-trapping agent. For ·O_2_^−^ detection, methanol was used as the dispersion medium, whereas water was used for ·OH detection; spectra were acquired in the dark and under visible-light illumination (λ ≥ 420 nm). For the spin-trapping measurements, the concentration of the DMPO trapping agent was fixed at 50 mM in the respective solvent matrices. In situ illumination was provided by a 300 W Xe lamp coupled with a highly focused optical fiber directed directly into the resonant cavity. The EPR acquisition parameters were standardized as follows: X-band microwave frequency ≈ 9.8 GHz, microwave power = 20.0 mW, and modulation amplitude = 1.0 G.

### 4.6. Photocatalytic Activity Evaluation

The same operating conditions were used for all catalysts to permit a direct comparison of their photocatalytic performance. The photocatalytic performance was evaluated under visible-light irradiation from a 350 W Xe arc lamp equipped with a 420 nm cutoff filter (λ ≥ 420 nm) using a home-made photoreactor [44]. The reactions were conducted in a custom-built cylindrical double-jacketed Pyrex glass photoreactor. The 350 W Xe arc lamp was positioned vertically at a fixed lamp–sample distance of approximately 10 cm, delivering a relatively uniform optical irradiance of ~100 mW cm^−2^ at the liquid surface. Furthermore, the suspension temperature was strictly maintained at 25.0 ± 0.5 °C throughout the entire reaction process via a continuous circulating cooling-water jacket, thereby rigorously excluding any confounding thermal catalytic effects.

In a typical run, 40 mg of catalyst was dispersed in 40 mL of a 10 mg·L^−1^ pollutant solution. The suspension was magnetically stirred in the dark for 60 min to reach adsorption–desorption equilibrium and then irradiated. At given intervals, about 4 mL of the suspension was withdrawn and centrifuged, and the residual pollutant concentration in the supernatant was determined from its absorbance at the maximum absorption wavelength on a UV–vis spectrophotometer. The degradation efficiency was calculated as η = (C_0_ − C_t_)/C_0_ × 100%, and the kinetics were fitted with the pseudo-first-order model ln(C_0_/C_t_) = kt [40,45]. The degradation of RhB and CIP was carried out under conditions identical to those for MB, except that the irradiation time was extended to 210 min for CIP. All photocatalytic experiments were independently performed in triplicate, and the results are presented as the mean ± standard deviation. Error bars represent the standard deviation of three independent measurements (*n* = 3).

For the radical-trapping experiments, AO, t-BuOH, and BQ were separately introduced into the MB suspension prior to illumination as scavengers for h^+^, •OH, and •O_2_^−^, respectively. The cycling stability was assessed over six consecutive RhB degradation runs; after each run, the monolithic catalyst was retrieved, washed with deionized water, dried, and reused.

## Figures and Tables

**Table 1:** Pseudo-first-order rate constants (k) and correlation coefficients (R^2^) for MB degradation over the AgX/CA photocatalysts.

Sample	k (min^−1^)	R^2^
AgBr	0.0147	0.9752
10 wt% AgBr/CA	0.0249	0.9904
20 wt% AgBr/CA	0.0321	0.9966
30 wt% AgBr/CA	0.0387	0.976
AgCl	0.0148	0.9841
10 wt% AgCl/CA	0.0211	0.9831
20 wt% AgCl/CA	0.0254	0.9893
30 wt% AgCl/CA	0.0325	0.9842

**Table 2:** Comparisons of the photocatalytic performance and operating conditions of the recently reported AgX-based photocatalysts.

Photocatalyst	Pollutant	Initial Concentration and Volume	Catalyst Dosage	Light Source	Dark Adsorption	Irradiation Time	Degradation Efficiency	Kinetic Constant	Ref.
Ag/AgBr/MgBi_2_O_6_	MB	15 mg L^−1^, 50 mL	150 mg; 3.0 g L^−1^	2.5 W white-light LED; 0.38 W cm^−2^	30 min	40 min	98.6%	0.1223 min^−1^	[13]
Ag_2_O/AgBr–CeO_2_	TC	10 mg L^−1^, 50 mL	25 mg; 0.5 g L^−1^	500 W Xe lamp, λ > 420 nm	30 min	60 min	≈93.7%	0.04014 min^−1^	[18]
Ag_3_BiO_3_/AgBr	RhB/MB	10^−5^ mol L^−1^, 50 mL	40 mg; 0.8 g L^−1^	300 W Xe lamp, visible light	NR	80 min	96%/100%	0.0366/0.0412 min^−1^	[26]
BiOBr/AgBr	BPA	NR	0.5 g L^−1^	Visible light, 400–780 nm	NR	180 min	76.6%	0.0069 min^−1^	[31]
AgBr/Ag/TiO_2_{100}	TC	20 mg L^−1^, 100 mL	20 mg; 0.2 g L^−1^	300 W Xe lamp, λ > 420 nm	30 min	120 min	82.5%	2.56 × 10^−3^ mg^−1^ L min^−1^	[32]
AgBr/BiFeO_3_	LR5B	40 mg L^−1^, 40 mL	40 mg; 1.0 g L^−1^	350 W Xe lamp, visible light	30 min	60 min	95.72%	0.03908 min^−1^	[39]
Ag/AgCl-NW/rGO	RhB	10 ppm, 50 mL, pH 8	30 mg; 0.6 g L^−1^	300 W halogen lamp, λ ≥ 420 nm; lamp–reactor distance 25 cm	30 min	120 min	99.78%	0.0498 min^−1^	[40]
30 wt% AgCl/CA	MB	10 mg L^−1^, 40 mL	40 mg; 1.0 g L^−1^	350 W Xe lamp, λ > 420 nm	60 min	60 min	91.71%	0.0325 min^−1^	This work
30 wt% AgBr/CA	MB	10 mg L^−1^, 40 mL	40 mg; 1.0 g L^−1^	350 W Xe lamp, λ > 420 nm	60 min	60 min	95.68%	0.0387 min^−1^	This work

Notes: RhB, rhodamine B; MB, methylene blue; BPA, bisphenol A; TC, tetracycline; LR5B, Lanasol Red 5B; NR, not reported. Normalized catalyst dosage was calculated by dividing the reported catalyst mass by the reaction-solution volume.

## Data Availability

The original contributions presented in the study are included in the article/Supplementary Material; further inquiries can be directed to the corresponding author.

## Supplementary Materials

The following supporting information can be downloaded at https://www.mdpi.com/article/10.3390/gels12080711/s1, Figure S1. XRD patterns of (a) the AgBr system (CA, 10–30 wt% AgBr/CA, and pristine AgBr) and (b) the AgCl system (CA, 10–30 wt% AgCl/CA, and pristine AgCl). Figure S2. DMPO–•OH ESR spectra of 30 wt% AgBr/CA in aqueous dispersion under dark and visible-light (λ ≥ 420 nm) conditions. Figure S3. Particle-size distributions obtained from TEM images of (a) AgBr nanoparticles in 30 wt% AgBr/CA and (b) AgCl nanoparticles in 30 wt% AgCl/CA. More than 100 nanoparticles were measured for each sample. Figure S4. Tauc plots used to estimate the optical band gaps of pristine (a) AgBr and (b) AgCl. Figure S5. XPS survey spectra of (a) 30 wt% AgBr/CA and (b) 30 wt% AgCl/CA. Figure S6. Time-resolved photoluminescence (TRPL) decay spectra. Figure S7. (a) N_2_ adsorption–desorption isotherms and (b) the corresponding pore size distribution curves of the pristine CA, 30 wt% AgBr/CA, and 30 wt% AgCl/CA composites. Figure S8. Post-cycling structural and morphological stability characterizations. Table S1. Comparison of relative contents of various elements on sample surfaces analyzed by XPS and EDS.
