# Supplementary material for: Monolithic AgX/Biomass Carbon Aerogels (X = Br, Cl) for Recyclable Photocatalytic Degradation of Multiple Pollutant Classes
Source: Gels. 2026 Aug 11;12(8):711. doi: 10.3390/gels12080711 (PMC13512214; doi:10.3390/gels12080711)
Supplement: Supplementary file 1 [file gels-12-00711-s001.zip › gels-4418179-supplementary.pdf]

Monolithic AgX/Biomass Carbon Aerogels (X = Br, Cl) for Recyclable Photocatalytic Degradation of Multiple Pollutant Classes

Ziyang Tang [1], Zhicheng Zhu [1], Xihao Sun [1], Yuxin Sun [1], Bencong Zhang [1], Mingmei Zhang [1], Jialu Lu [2] and Wei Wei [1]*

[1] School of Chemistry and Chemical Engineering, Public Experiment and Service Center, Jiangsu University, Xuefu Road 301, Zhenjiang 212013, China

[2] Shanghai Key Laboratory of Special Artificial Microstructure and Technology, School of Physics Science and Engineering, Tongji University, Siping Road 1239, Shanghai 200092, China; jialu21@tongji.edu.cn

* Correspondence: weiwei@ujs.edu.cn

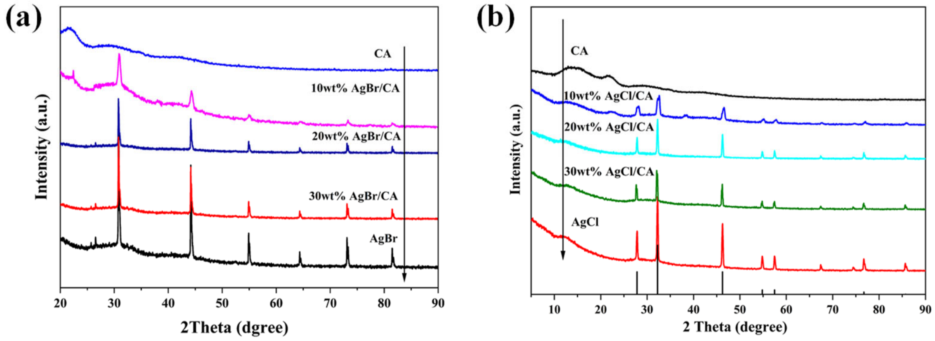

Figure S1. XRD patterns of (a) the AgBr system (CA, 10–30 wt% AgBr/CA, and pristine AgBr) and (b) the AgCl system (CA, 10–30 wt% AgCl/CA, and pristine AgCl).

The intensities of the characteristic silver-halide reflections increase progressively with the AgX loading, while the patterns remain consistent with cubic AgBr (JCPDS No. 79-0149) and cubic AgCl (JCPDS No. 31-1238), respectively, confirming the phase purity of the composites across the whole loading range.

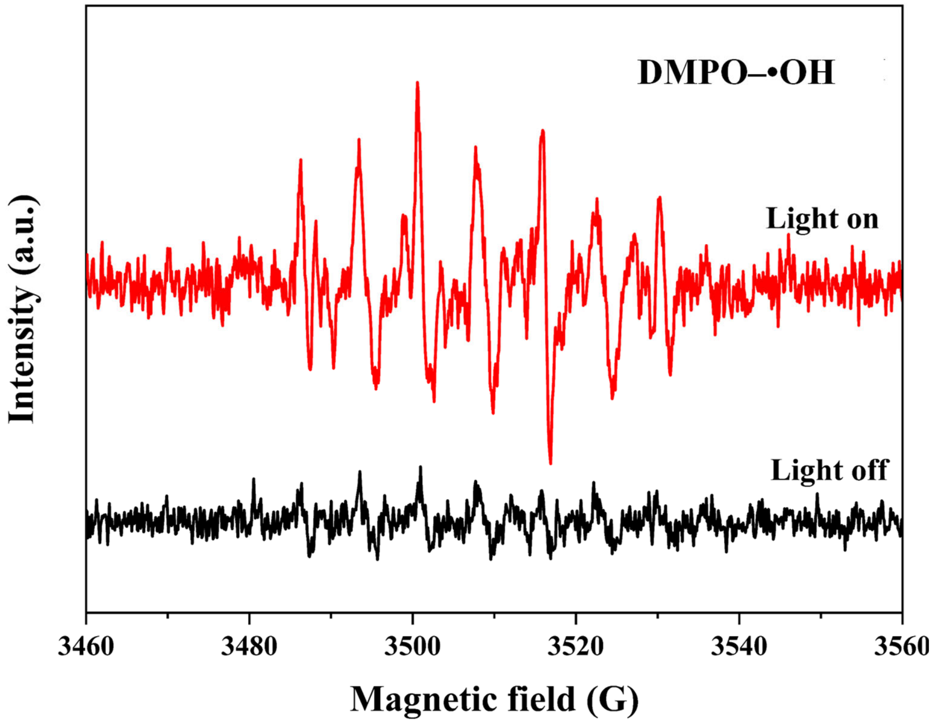

Figure S2. DMPO–•OH ESR spectra of 30 wt% AgBr/CA in aqueous dispersion under dark and visible-light ($\lambda \geq 420$ nm) conditions.

The characteristic DMPO–•OH signal appears only under illumination, providing direct evidence for the photoinduced generation of hydroxyl radicals over AgBr/CA.

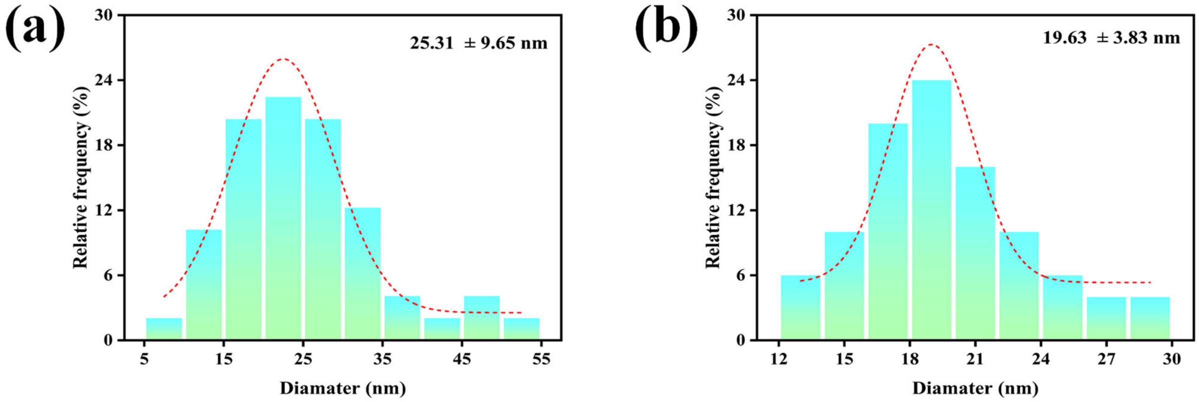

Figure S3. Particle-size distributions obtained from TEM images of (a) AgBr nanoparticles in 30 wt% AgBr/CA and (b) AgCl nanoparticles in 30 wt% AgCl/CA. More than 100 nanoparticles were measured for each sample.

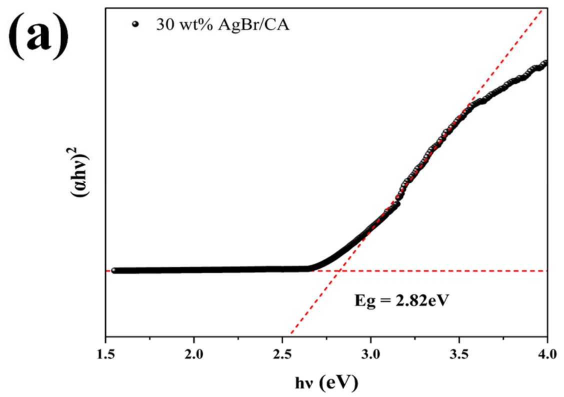

Figure S4. Tauc plots used to estimate the optical band gaps of pristine (a) AgBr and (b) AgCl. The plots were constructed assuming direct allowed transitions by plotting $[\alpha h\nu]^2$ as a function of hν, giving estimated band-gap values of approximately 2.82 and 3.04 eV for AgBr and AgCl, respec-tively.

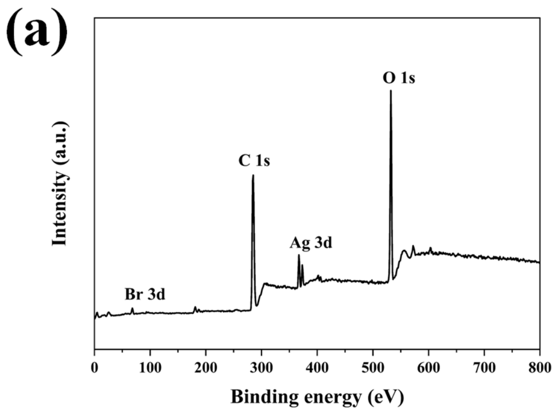

Figure S5. XPS survey spectra of (a) 30 wt% AgBr/CA and (b) 30 wt% AgCl/CA.

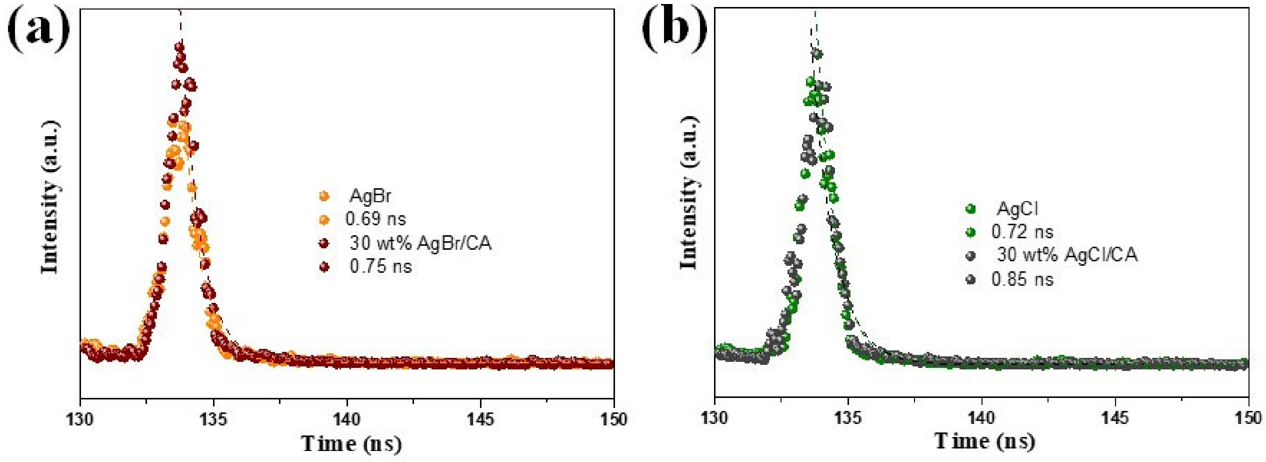

Figure S6. Time-resolved photoluminescence (TRPL) emission decay spectra of (a) pristine AgBr and 30 wt% AgBr/CA, and (b) pristine AgCl and 30 wt% AgCl/CA. The calculated average charge-carrier lifetimes (τ) are indicated in the respective panels.

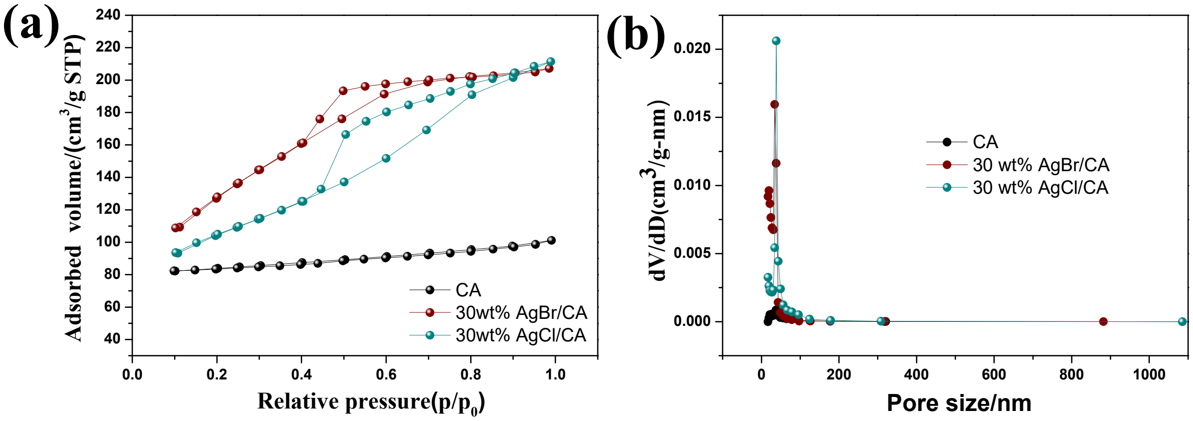

Figure S7. (a) N_2 adsorption-desorption isotherms and (b) the corresponding pore size distribution curves of the pristine CA, 30 wt% AgBr/CA, and 30 wt% AgCl/CA composites.

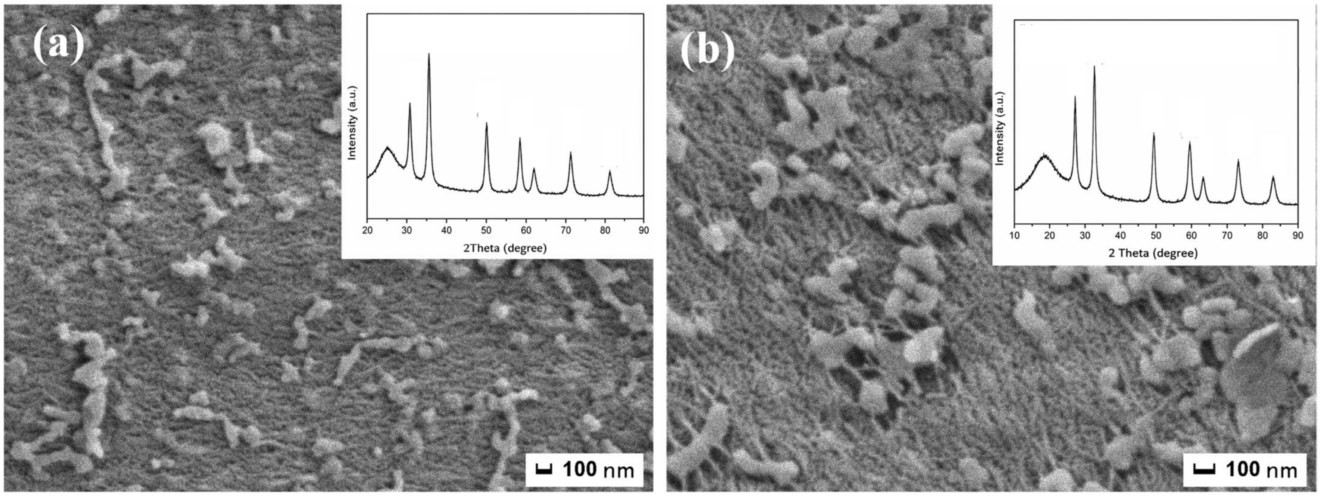

Figure S8. Post-cycling structural and morphological stability characterizations. Post-reaction SEM image and the corresponding XRD pattern (inset) of the retrieved (a) 30 wt% AgBr/CA and (b) 30 wt% AgCl/CA composites after six consecutive photocatalytic degradation cycles.

Table S1. Comparison of relative contents of various elements on sample surfaces analyzed by XPS and EDS.

	30 wt% AgBr/CA			30 wt% AgCl/CA	
Element	Atomic percentage/% XPS	Atomic percent (at%) EDS	Element	Atomic percentage/% XPS	Atomic percent (at%) EDS
O	29.63	22.62	O	30.64	28.18
C	68.37	75.02	C	67.99	70.23
Ag	0.86	1.05	Ag	0.75	0.76
Br	1.05	1.31	Cl	0.62	0.83
